# Bubonic Plague

**Published:** 1915-10

**Authors:** 


**Bubonic Plague.** J. Guiteras, Sanidad & Beneficencia, Havana, November and December, 1914.

Guiteras describes the last outbreak of the plague in Havana. He calls attention to the conveyance of fleas, infected and otherwise in sacking. He maintains that this is a frequent means of conveyance but only for short distances. An infected rat will, when it dies, leave a number of fleas in the sacking where these insects find, for the time being, a favorable resort; and the conveyance of them in sacks can be readily imagined. In the course of days they lose their infectivity and become disseminated besides; so that the charge of infection does not reach the new locality in concentrated form. The possibility of the transmission of the infection must diminish in proportion with the distance of transport in time and space.

To test the infectivity of any given locality, he allows guinea pigs to remain at the site for ten days, keeps them under observation for seven days, when they are killed and examined macroscopically and bacteriologically for evidence of the disease.

Of interest is the fact that no cases of plague were found in tobacco warehouses or those containing charcoal. In one of his experiments Guiteas divided a breeding jar for fleas by a low partition, on one side of which he placed ordinary earth and one the other earth mixed with charcoal dust. The fleas placed therein speedily deserted the side containing the charcoal dust.

While one is in bed the fleas are as likely to bite the upper as the lower part of the body but while at work it is the lower extremities that suffer—hence the frequency of groin and femoral buboes. The sanitary workers are therefore compelled to wear boots which have been rubbed with crude petroleum and this has proved to be an effectual means to avoid infection.

The value of fumigation has been questioned, for one effect seems to be the driving of rats from the infected locality with a consequent spread of the disease.

The free use of water in infected dwellings is of undoubted value—not with the idea of drowning the rats but to thoroughly wet them and their runs and thus seriously affect the breeding of the fleas.

He attributes the low mortality of the last epidemic to the good sanitary organization and the reporting of all cases, light and severe, rather than by the assumption that in recent years the great epidemic diseases, plague, cholera and yellow fever have become attenuated.

